# Mechanisms of Virus-Induced Airway Immunity Dysfunction in the Pathogenesis of COPD Disease, Progression, and Exacerbation

**DOI:** 10.3389/fimmu.2020.01205

**Published:** 2020-06-16

**Authors:** Hong Guo-Parke, Dermot Linden, Sinéad Weldon, Joseph C. Kidney, Clifford C. Taggart

**Affiliations:** ^1^Airway Innate Immunity Research Group, Wellcome Wolfson Institute for Experimental Medicine, School of Medicine, Dentistry & Biomedical Sciences, Queens University Belfast, Belfast, United Kingdom; ^2^Department of Respiratory Medicine Mater Hospital Belfast, Belfast, United Kingdom

**Keywords:** chronic obstructive pulmonary disease, virus, inflammation, infection, lung damage, acute pulmonary exacerbation

## Abstract

Chronic obstructive pulmonary disease (COPD) is the integrated form of chronic obstructive bronchitis and pulmonary emphysema, characterized by persistent small airway inflammation and progressive irreversible airflow limitation. COPD is characterized by acute pulmonary exacerbations and associated accelerated lung function decline, hospitalization, readmission and an increased risk of mortality, leading to huge social-economic burdens. Recent evidence suggests ~50% of COPD acute exacerbations are connected with a range of respiratory viral infections. Nevertheless, respiratory viral infections have been linked to the severity and frequency of exacerbations and virus-induced secondary bacterial infections often result in a synergistic decline of lung function and longer hospitalization. Here, we review current advances in understanding the cellular and molecular mechanisms underlying the pathogenesis of COPD and the increased susceptibility to virus-induced exacerbations and associated immune dysfunction in patients with COPD. The multiple immune regulators and inflammatory signaling pathways known to be involved in host-virus responses are discussed. As respiratory viruses primarily target airway epithelial cells, virus-induced inflammatory responses in airway epithelium are of particular focus. Targeting virus-induced inflammatory pathways in airway epithelial cells such as Toll like receptors (TLRs), interferons, inflammasomes, or direct blockade of virus entry and replication may represent attractive future therapeutic targets with improved efficacy. Elucidation of the cellular and molecular mechanisms of virus infections in COPD pathogenesis will undoubtedly facilitate the development of these potential novel therapies that may attenuate the relentless progression of this heterogeneous and complex disease and reduce morbidity and mortality.

## Introduction

Chronic obstructive pulmonary disease (COPD) is the umbrella term for chronic obstructive bronchitis and pulmonary emphysema, and is characterized by persistent small airway inflammation and progressive irreversible airflow limitation (1–5). COPD is associated with acute pulmonary exacerbations, accelerated lung function decline and increased risk of mortality (6, 7). As a common global epidemic, COPD affects 10% of the population and is the third leading cause of death worldwide (3). Viral and bacterial infections are key elements in the pathogenesis of exacerbations (5–9). Recent evidence suggests respiratory viral infections cause ~50% of COPD acute exacerbations (5, 6, 10). Secondary bacterial infections often ensue with pronounced illness (6).

However, the underlying mechanisms of how viruses subvert host immune defense systems in COPD exacerbations are not completely understood. Herein, we review current advances in understanding the cellular and molecular mechanisms associated with the increased susceptibility to virus infections. As respiratory viruses preferentially infect airway epithelial cells, we focus on virus-induced inflammatory responses in airway epithelium. Understanding these pathogenic pathways may facilitate the development of potential novel therapies to attenuate the relentless progression of the disease.

## COPD Pathogenesis

Cigarette smoking is the predominant etiologic factor in the development of COPD (3–5). Other risk factors include host genetic factors, which is most evident in alpha-1 antitrypsin (AAT) deficiency (11–13). Recently, childhood respiratory viral infections have been postulated as an independent risk factor associated with COPD later in life (14). Other environmental factors such as pollutant and occupational exposure to dusts or fumes, particularly organic dusts are strongly associated with COPD (4, 13, 15, 16). Social deprivation is also a factor in the development of COPD (6, 17, 18).

Cigarette smoke and other inhale noxious gases induce an abnormal inflammatory response, that is further amplified by protease and oxidative stress, which are central to COPD pathogenesis (8, 11). Persistent small airway inflammation and the resulting destruction of the lung architecture leads to emphysema and loss of lung elastic recoil, chronic bronchitis induced mucus hypersecretion and airflow obstruction, as well as peribronchial fibrosis (11, 19, 20). Excessive neutrophilic infiltration and associated proteolytic enzymes including neutrophil elastase are hallmark features of smoke-induced inflammation (19, 21–25). Consequently, the protease/antiprotease imbalance contributes to the pathogenesis of emphysema due to the increased breakdown of elastin and loss of elastic recoil in the lung parenchyma (19, 21–24). Diminished activity of protein phosphatase 2A (PP2A), a regulator of the inflammatory response in the airways, has been demonstrated in COPD and upregulation of PP2A activity can ameliorate inflammation in a cigarette smoke model of COPD by reducing activity of the cysteine protease, cathepsin S (26). Recent research has proposed a role for formylated peptides and formyl peptide receptor (FPR) receptor signaling in the initiation and progression of lung disease in current and former smokers (27, 28). These peptides are present in tobacco leaves and are actively secreted by bacteria or passively released from dead and dying host cells after tissue injury (29). FPR1 and FPR2 activation may play a role in neutrophil migration, degranulation, reactive oxygen species (ROS) production, and phagocytosis (29, 30). A novel cross-talk mechanism was identified in neutrophils, by which signals generated by the purinergic receptor for ATP (P2Y_2_) reactivate ligand-bound inactive FPRs, which resume signaling (31). Furthermore, a role for purinergic receptors in the pathophysiology of COPD has been demonstrated in human and experimental models (32–35), however, further work is needed to elucidate its role in the immune dysfunction associated with COPD (36). Excessive production of ROS results in an oxidant-antioxidant imbalance leading to oxidative stress and is a major predisposing feature in the development of the disease (37–41). Therefore, a vicious cycle is created in which inflammation drives a protease-antiprotease and oxidant-antioxidant imbalance, as well as multiple intracellular cell signaling mechanisms, which potentiate inflammation, goblet cell hyperplasia and mucus hypersecretion (8, 40).

Chronic low-grade respiratory syncytial virus (RSV) infection has also been implicated in COPD pathogenesis (42–45). However, the detection of RSV infection in stable COPD remains controversial (46, 47). Hogg and colleagues showed that the E1A region of the adenovirus may contribute to COPD pathogenesis by enhancing soluble ICAM-1 expression and inflammatory cells infiltration (48). In contrast, another study failed to demonstrate the persistent presence of adenovirus V or E1A (49). Polosukhin at al. detected Epstein Barr Virus (EBV) positive cells in COPD lung tissue sections by immunochemistry staining (50). Consistent with this finding, we have demonstrated that EBV DNA is frequently present in COPD sputum compared with unaffected smokers (51). Latent viral infections and cigarette smoke may synergistically contribute to the chronic inflammation in COPD (52). COPD is a heterogeneous disease with a complex etiology, however, acute and chronic lower respiratory tract infections occur with increased frequency in patients with COPD. Whatever the cause, it is clear that a defective host response plays an important role and improving our understanding of the mechanisms involved is essential to improving prevention and treatment strategies.

## Airway Epithelium Dysfunction in COPD

**Figure 1:**
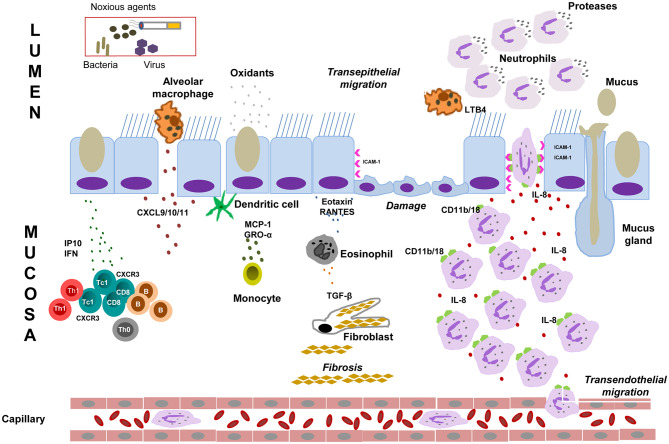
Mechanisms of airway immunity dysfunction in COPD. Cigarette smoke and noxious agents activate epithelial cells and macrophages to release chemotactic factors such as CXCL9 (MIG), CXCL10 (IP10), and CXCL11 (I-TAC), which increase CD8+ T cells, dendritic cells, B lymphocytes and eosinophil infiltration into the airway mucosa. These inflammatory cells together with macrophages and epithelial cells initiate an inflammatory cascade that triggers the release of inflammatory mediators such as TNF-α, IFN-γ, proteases (such as MMPs), inflammatory cytokines and chemokines (IL-1, IL-6, IL-8) and growth factors. These inflammatory mediators sustain the airway mucosal inflammatory process in COPD, which cause elastin degradation and emphysema. Epithelial cells and macrophages also release TGF-β, which stimulates fibroblast proliferation resulting in small airway fibrosis. During exacerbation, the inflammatory burden in the small airways over-powers host anti-inflammatory mechanisms leading to profound alveolar damage and inflammation. Cigarette smoke and other irritants activate epithelial cells and macrophages to release neutrophil chemoattractants, such as LTB4, IL-8, TNFα, CXC chemokines (MCP-1, GRO-α, and GM-CSF). CXC chemokines also act as chemoattractants for monocytes. Cigarette smoke causes increased level of ROS produced in the airways is reflected by increased markers of oxidative stress. Oxidative stress is involved in several events in the pathogenesis of COPD including oxidative inactivation of anti-proteases and surfactants, mucus hypersecretion, alveolar epithelial injury, remodeling of extracellular matrix and apoptosis. Neutrophils bind to ICAM-1, the level of which has been found to upregulated in bronchial epithelial cells in COPD. Neutrophils then migrate to the respiratory tract under the control of IL8/LTB4 chemotactic gradient. These cells then release proteases that break down connective tissue in the lung parenchyma, resulting in emphysema. Neutrophil elastase release in airway induces epithelial barrier dysfunction, mucus hypersecretion and reduces mucociliary clearance. GM-CSF, Granulocyte-macrophage colony-stimulating factor: GRO-α, Growth-regulated oncogene-α; ICAM-1, epithelial intercellular adhesion molecule-1; LTB4, leukotriene B4; IL, interleukin; IP10, CXCL10, interferon g-induced protein 10; I-TAC, CXCL11, interferon-inducible T-cell α chemoattractant; MCP-1, monocyte chemoattractant protein-1; MIG, CXCL9, monokine induced by g interferon; MMPs, matrix metalloproteinases; RANTES, regulated on activation, normal T cell expressed and secreted; ROS: reactive oxygen species; TGF, transforming growth factor; TNF-α, Tumor necrosis factor-α; IFN, interferon.

Normal airway epithelial cells play a pivotal role in innate immune defense. They act as a barrier to pathogens and noxious stimuli and produce mediators and enzymes to orchestrate and maintain proper functioning of the innate and adaptive immune responses (24, 53, 54). As illustrated in Figure 1, the COPD airway epithelium responds to cigarette smoke by secreting inflammatory mediators and recruiting immune cells to the site of damage to orchestrate the inflammatory response. A robust infiltration of macrophages and CD8^+^ T cells, and to a lesser extent CD4^+^ T cells, in the airway mucosa as well as elevated neutrophils in the airway lumen are the hallmark features of COPD inflammation, the degree of which correlates to disease severity (46, 55). Increased levels of epithelial-derived CXCL9 (MIG), CXCL10 (IP-10), and CXCL11 (I-TAC) and their receptor CXCR3 has been demonstrated to contribute, in part, to the mechanism of CD8^+^ cellular accumulation (40, 53, 54). CD8^+^ T cells release IP-10, TNF-α, IFN-γ, perforins, and granzyme, and have been associated with alveolar epithelial cell apoptosis (19, 37, 56, 57). As COPD progresses, elevated numbers of dendritic cells and B lymphocytes also appear in the airways and alveolar walls. CD8^+^ T cells and B cells organize into lymphoid follicles and may contribute to increased “immune surveillance” in COPD (19, 37, 39). The airway epithelium also releases a cascade of secondary mediators including cytokines, lipid mediators, growth factors, proteases, antiproteases and ROS to escalate COPD inflammation (53, 54, 58). Cigarette smoke and other irritants also activate epithelial cells and macrophages to release neutrophil and macrophage chemoattractants, such as LTB4, IL-8, and related CXC chemokines (MCP-1, GRO-α and GM-CSF), which contribute to the development of emphysema (39, 46, 59, 60).

The mechanism of neutrophilic inflammation has been linked to CD11b/CD18 on neutrophils binding to ICAM-1 on bronchial epithelium, which is up-regulated in COPD (54, 61–63). Neutrophils migrate to the respiratory tract and release serine proteases, matrix metalloproteinases (MMPs) and oxidants (24, 40, 46). Neutrophil serine proteases are associated with emphysema, mucus hypersecretion, increased risk of exacerbation and accelerated forced expiratory volume in 1 s (FEV_1_) decline (64–66). Subsequently, these proteases degrade extracellular matrix components leading to the destruction of the alveolar wall, epithelial barrier dysfunction, reduction in mucociliary clearance, mucus hypersecretion and goblet cell metaplasia through activation of the epidermal growth factor receptor (EGFR) (37, 59, 64). Moreover, alveolar epithelial cells also secrete transforming growth factor-β (TGF-β) which may contribute to small airway fibrosis and emphysema (67).

## Molecular Mechanisms Associated With Viral-Induced COPD Exacerbations

### Viral-Induced COPD Exacerbations

Acute exacerbations of COPD are characterized by a sudden decline in lung function, hospitalization and high mortality (7, 9, 46). The complicated interaction between the host and viral or bacterial infections or co-infection, as well as environmental factors, precipitate the onset of exacerbations. These factors amplify the inflammatory burden in the small airway, overpowering host anti-inflammatory mechanisms leading to profound airway obstruction in COPD (46, 68–70). Severe virus-associated exacerbations also induce elevated levels of CD8^+^ T cells, neutrophils, eosinophils, TNF-α and IL-6 in the sputum of COPD patients (68–70).

Exacerbations often occur seasonally accompanied by common cold-like symptoms implicating respiratory viral infections rather than hitherto suspected bacterial infection (43, 44). Respiratory virus infection, including human rhinovirus (HRV), influenza virus (IAV), coronavirus, RSV, human parainfluenza, metapneumovirus (hMPV) and adenovirus initiate nearly 50% of COPD exacerbations often with more severe symptoms (69–73). Viruses have developed a myriad of aversion strategies to subvert and manipulate host immune responses and these have been recently reviewed elsewhere (74, 75). Most respiratory viruses target airway epithelial cells leading to epithelial barrier destruction, microvascular dilatation, oedema and immune cell infiltration (58, 70–72). These viruses are associated with small airway secondary bacterial infection, thus magnifying the inflammatory response in COPD leading to a synergistic deterioration in lung function and prolonged hospitalization (42, 44, 71).

As detailed below, recent research has focused on immune regulators and inflammatory signaling pathways orchestrating the underlying mechanisms of increased susceptibility to virus-associated exacerbation and the exaggerated inflammatory response in COPD airways and potential therapeutic inventions.

### T Cell Exhaustion

Although accumulated CD8^+^ T cells are present in greater numbers in severe COPD, a diminished CD8^+^ T cell antiviral response, worsened airflow limitation and respiratory symptoms have been reported in IAV and HRV-induced COPD exacerbations (68, 71, 76, 77). As a result, CD8^+^ cells potentially amplify airway epithelium destruction and promote tissue injury through mechanisms including direct cytotoxic effects, pro-inflammatory signaling and recruitment of other immune cells, leading to increased susceptibility to virus infections of airway epithelium (42–44, 69).

In COPD, prolonged receptor–ligand interaction during T cell activation may be linked to T cell exhaustion. McKendry and colleagues investigated increased CD8^+^ activation through the programmed cell death protein (PD)-1 exhaustion pathway as a potential mechanism of viral-induced COPD exacerbations (76). Dysregulation of T-cell cytotoxicity was associated with elevated levels of PD-1, which further increased following influenza infection in COPD patients (76). In contrast, infection-induced expression of the ligand PD-L1 on COPD macrophages was diminished, with a concomitant increase in IFN-γ release. These synergistic effects may cause excessive T-cell inflammation in response to virus infection.

### The NF-κB Pathway

The NF-κB pathway is consistently activated in COPD macrophages and airway epithelium, in particular, during bacterial or viral infections (78). Upon pathogen stimulation, the canonical pathway is mainly triggered by Toll like receptors (TLRs) and pro-inflammatory cytokines such as TNFα and IL-1 leading to the activation of the RelA containing NF-κB complexes. This initiates the translocation of RelA (p65)/p50 to the nucleus, where it induces the transcriptional response of pro-inflammatory and cell survival genes (78–80). The alternative non-canonical NF-κB pathway signals through a subset of receptors to activate the kinase NIK and IKKα complexes and downstream NF-κB2 p100 leading to the p52/RelB nucleus translocation and lymphoid organogenesis and B cell activation (78, 79).

Persistent or prolonged activation of NF-κB may contribute to COPD pathogenesis by switching on the transcriptional response of pro-inflammatory cytokines, chemokines, cell adhesion molecules (CAMs), proteases, and inhibitors of apoptosis to amplify inflammation. Therefore, strategies, which block the activation of NF-κB, offer attractive therapeutic options to regulate COPD inflammation. Several IKK-β inhibitors have been identified to inhibit p65 nuclear translocation and exert anti-inflammatory effects (81, 82). Lung-targeted overexpression of RelB has also been demonstrated to protect against cigarette smoke–induced inflammation by reducing inflammatory mediator production (83). In COPD airway epithelium, influenza virus infection increased microRNA-125a/b, which directly inhibits A20 and mitochondrial antiviral-signaling protein (MAVS) to promote inflammation and impair antiviral responses in COPD (84). Thus, miR-125a/b may provide a potential therapeutic target for both inflammation and antiviral responses in COPD.

### TLR Sensing and EGFR Signaling

**Figure 2:**
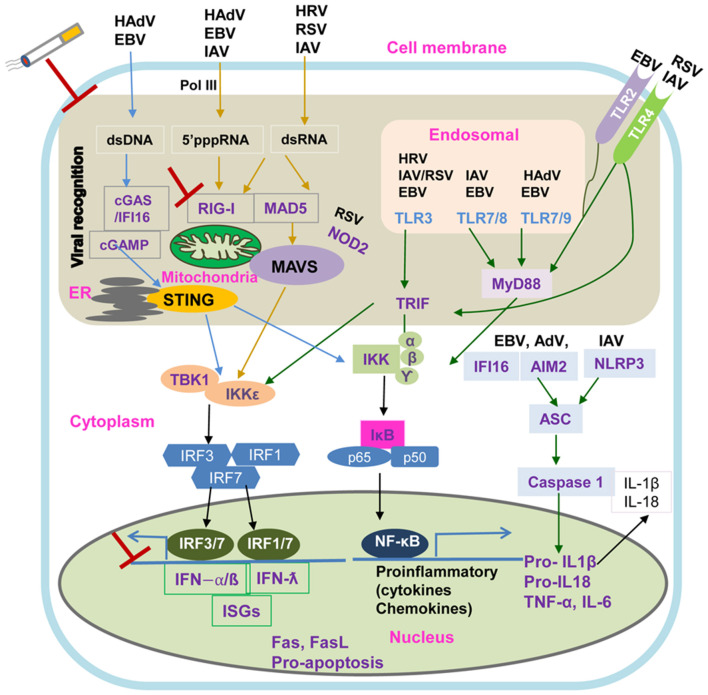
Intracellular Viral Sensing Pathways. DNA and RNA viruses release their genomes in the cytoplasm, where host innate sensors for nucleic acids reside. Upon ss/dsRNA binding, RIG-I engages the adaptor protein MAVS on the mitochondrial outer membrane. The cGAS receptor recognizes dsDNA and the RNA:DNA hybrids generated during retroviral replication and catalyzes the synthesis of cGAMP, which is the primary agonist of the adaptor protein STING. Another sensor, IFI16 can recruit STING in response to cytoplasmic DNA through a molecular mechanism yet to be described. Both STING and MAVS stimulate downstream signaling cascades that involve multiple kinases and finally lead to IRF3 phosphorylation and nuclear translocation. The primary consequence of these virus sensing pathways is the induction of type I IFN and IFN stimulated genes. cGAS, cyclic GMP-AMP synthase; cGAMP, 2′3′guanosine-adenosine monophosphate; IFI16, interferon-g inducible protein 16; IKK, IkB kinase; IRF3, interferon regulatory factor 3; MAVS, mitochondrial antiviral-signaling protein; RIG-I, retinoic acid inducible gene-I; ss/dsRNA, single-stranded/double-stranded RNA; vRNA/DNA, viral RNA/DNA; STING, stimulator of interferon genes; TANK, TRAF-associated NF-kB activator; TBK1, TANK binding kinase 1.

Figure 2 illustrates key virus innate recognition signaling pathways in COPD airway epithelium. Briefly, ssRNAs of HRV, RSV, and IAV are recognized by TLR3 in the endosomes which consequently activate IRF-3 via the Toll/IL-1 receptor domain-containing adaptor (TRIF), leading to the induction of IFN-β and IFN-λ1. Other endosomal TLRs (TLR7/8 and TLR7/9) recognize the dsRNAs of IAV and adenovirus through MyD88-dependent pathway to activate NF-κB and IRF-7 to secrete pro-inflammatory mediators and IFNs, respectively. TLR4 expressed on the cell surface senses RSV and IAV, signaling through both the MyD88 and TRIF pathways to activate NF-κB and IRF-7. The airway epithelium may recognize EBV by endosomal TLRs and TLR2 at the cell surface to activate downstream pathways (85, 86). As a risk factor for RSV-induced COPD exacerbations, TLR3 activation has been found to correlate with lung function deterioration during exacerbations highlighting TLR3 blockade as a therapeutic target (87). However, Silkoff et al. showed that TLR3 inhibition was inefficient in attenuating HRV-induced experimental asthma exacerbation (88).

Many TLRs recognize pathogen-associated molecular patterns (PAMPs) to activate airway epithelial EGFR signaling cascades. Aberrant EGFR signaling promotes progressive lung fibrosis and mucus hypersecretion; characteristic features of COPD, asthma and cystic fibrosis pathogenesis (24, 89). The EGFR cascade consists of multiple receptors and extracellular ligands that function via receptor auto-phosphorylation and cytoplasmic protein binding of four downstream complexes including the mitogen-activated protein kinases/extracellular signal–regulated kinases (MEK/ERK), phosphatidylinositol 3-kinases/protein kinase B (PKB) (PI3K/AKT), Just Another Kinase/signal transducer and activator of transcription (JAK/STAT) and mammalian target of rapamycin (mTOR) pathways (89). In a murine COPD model, EGFR activation through PI3K inhibited ciliated cell apoptosis and allowed IL-13 to stimulate the trans-differentiation of ciliated to goblet cell metaplasia (90). HRV infection induced the phosphorylation of PKD, a downstream kinase of PI3K. PKD inhibitors have been reported to effectively block HRV, poliovirus (PV) and foot-and-mouth disease virus (FMDV) replication at an early stage of infection, highlighting the potential of PKD inhibition in anti-HRV therapy in COPD (91). Chronic inflammation can also induce ICAM-1 and its ligand fibrinogen has been shown to promote EGFR-dependent mucin production in the airways of subjects with mucus hypersecretion (92).

EGF and the EGFR ligand, TGF-α, have been reported to directly enhance TNF-α-induced IL-8 secretion in airway inflammation (93). Ganesan et al. found that abnormal EGFR activation contributed to enhanced IL-8 expression in COPD airways via the NF-κB regulator, FoxO3A (94). Interestingly, TLR3 also induced EGFR activation and EGFR ligands (TGF-α and amphiregulin), which in turn promote EGFR-ERK signaling and mucin production through an autocrine/paracrine loop (95). Collectively, TLR antiviral defense mechanisms integrate with the EGFR mediated epithelial proliferation/repair pathways and may play an important role in viral-induced airway remodeling and airway disease exacerbations (93, 95, 96).

Viral infection *per se* also activates EGFR and EGFR signaling to ERK1/2, while STATs control the severity of HRV mediated airway inflammation. *In vitro*, HRV induced goblet cell hyperplasia was demonstrated to function through NF-κB-dependent MMP-mediated TGF-α release, leading to EGFR activation and mucus secretion (97). Interestingly, virus-induced EGFR activation suppressed interferon regulatory factor 1 (IRF1)-dependent IFN-λ airway epithelial antiviral signaling (98, 99). Inhibiting virus-mediated EGFR signaling augmented IRF1, IFN-λ secretion and viral clearance, indicating EGFR pathways as potential therapeutic targets in viral-induced COPD exacerbations (99).

### Cytoplasmic-Sensing Pathways

As shown in Figure 2, the airway epithelium also detects viral invasion through cytoplasmic pathogen recognition receptors. DNA and RNA viruses release their genomes into cytoplasm, which are detected by the host through cytoplasmic retinoic acid-inducible gene I/melanoma differentiation-associated protein 5- mitochondrial antiviral-signaling protein (RIG-I/MDA5–MAVS) RNA-sensing and the cyclic GMP–AMP synthase- signaling effector stimulator of interferon genes (cGAS–STING) DNA-sensing pathways, respectively (100). Upon ss/dsRNA binding, the RNA helicases, RIG-I and MDA5, interact with the adaptor protein MAVS on the mitochondrial outer membrane to activate the downstream signaling of type I interferon antiviral responses (100, 101). In contrast, the cGAS receptor senses retroviral replication products, dsDNA and RNA/DNA hybrids, to induce the synthesis of cGAMP which binds and activates STING (100). Interferon γ-inducible protein 16 (IFI16), a novel DNA sensor, has been found to recruit STING to activate type I IFN signaling through an unknown molecular mechanism (102). STING and MAVS also stimulate downstream multiple kinase signaling cascades resulting in IRF3 phosphorylation and NF-κB nuclear translocation (101, 102).

The primary consequence of these virus-sensing pathways is the induction of type I/type III IFNs and IFN stimulated genes as well as the production of inflammatory cytokines and chemokines. Attenuation of the IFN response following virus infection could result in uncontrolled viral replication and an escalated inflammatory response, a potential mechanism of virus-induced exacerbations in COPD. IFNα/β deficiency has been demonstrated in bronchial biopsies of asthmatic patients with rhinovirus-induced exacerbations and smoking-induced COPD (103). Farazuddin et al. have demonstrated that quercetin, a potent antioxidant and anti-inflammatory agent with antiviral properties, effectively mitigates rhinovirus-induced COPD exacerbation in a mouse model (104). Elevated ICAM-1 expression on the surface of airway epithelium has been directly linked to the mechanism of increased susceptibility of HRV-induced acute exacerbation. As the receptor of the major group of HRV and a ligand of lymphocyte function-associated antigen 1 (LFA-1) on neutrophils, ICAM-1 over-expression has been shown on epithelial cells in smokers and patients with COPD (63, 105, 106). Blocking ICAM-1 may also represent as a potential therapeutic option in HRV-induced exacerbations.

### Direct Targeting of Viral Binding, Entry, and Replication

Strategies that directly prevent virus binding, entry and replication may provide attractive alternatives in the treatment of COPD exacerbations (107). Capsid binders represent attractive potential inhibitors of HRV entry, however, they are strain-specific and have shown no effect on improving lung function and exacerbation in clinical trials to date (106). Mousnier and colleagues demonstrated that a dual inhibitor of human N-myristoyltransferases NMT1 and NMT2 can inhibit host-cell N-myristoylation and completely prevent rhinoviral replication, highlighting the therapeutic potential of targeting myristoylation in blocking rhinovirus infection in COPD (108). Short palate, lung, nasal epithelium clone 1 (SPLUNC1), a multifunctional host defense protein, was demonstrated to inhibit IAV binding and entry into airway epithelial cells, indicating an antiviral role for this protein in the airways (109). Therefore, in the COPD lung, SPLUNC1 degradation by proteases such as neutrophil elastase and/or inactivation by cigarette smoke may increase susceptibility to viral as well as bacterial infections, in addition to airway dehydration (110, 111). Recent research suggests that, in addition to modulating neutrophil chemotaxis, FPR2 signaling may be an important player in viral replication and IAV pathogenesis (30, 112, 113).

### Inflammasome

The inflammasome is a multiprotein pro-inflammatory complex and serves as an important link between the innate and adaptive immune responses. Inflammasomes that are activated by IAV RNA, EBV and adenoviral DNA include the nucleotide binding and oligomerization domain (NOD)-like receptor family pyrin domain-containing 3 (NLRP3) protein, absent in melanoma 2 (AIM2) protein and IFI16 protein (114). The inflammasome complexes assemble after recognition of PAMPs or danger-associated molecular patterns (DAMPs) induced by virus-killed cells or tissue damage and interact with apoptosis-associated speck like protein containing a caspase recruitment domain (ASC) via caspase activation and recruitment domains (CARD)-CARD/caspase-1 pathway (115–117). Activation of the inflammasome complex results in the autocatalytic cleavage of caspase-1 and ultimately leads to the production of pro-inflammatory cytokines including IL-1β, IL-18 and pro-IL-33 (116, 117). Upon maturation, these cytokines mediate inflammatory responses by activating lymphocytes and facilitating their infiltration to the site of primary infection and by inducing IFNs and other pro-inflammatory cytokines secretions (116).

## Concluding Remarks

COPD is a heterogeneous and complex disease resulting from the deregulation of multiple immune regulators and inflammatory signaling pathways. Significant progress has been made to elucidate the causative mechanism of COPD pathophysiology including viral infection in disease development, severity and exacerbations. Targeting virus-induced inflammatory pathways such as T cell exhaustion, NF-κB, TLRs, EGFR, interferons and the inflammasome provide attractive future therapeutic options. Understanding the cellular and molecular mechanisms of virus-induced COPD pathogenesis could potentially limit pathogen-mediated disease exacerbations and minimize viral-associated inflammation, tissue destruction and pulmonary function deterioration.

## Author Contributions

All authors listed have made a substantial, direct and intellectual contribution to the work, and approved it for publication.

## Conflict of Interest

JK was a non-executive director and shareholder of Hibergene Diagnostics Ltd. The remaining authors declare that the research was conducted in the absence of any commercial or financial relationships that could be construed as a potential conflict of interest.
